# Cloning of a gene (SR-A1), encoding for a new member of the human Ser/Arg-rich family of pre-mRNA splicing factors: overexpression in aggressive ovarian cancer

**DOI:** 10.1054/bjoc.2001.1885

**Published:** 2001-07

**Authors:** A Scorilas, L Kyriakopoulou, D Katsaros, E P Diamandis

**Affiliations:** Department of Pathology and Laboratory Medicine, Mount Sinai Hospital, Toronto, Ontario, M5G 1X5, Canada; Department of Laboratory Medicine and Pathobiology, University of Toronto, Toronto, Ontario, M5G 1L5, Canada; Department of Gynecology, Gynecologic Oncology Unit, University of Turin, Turin, 10126, Italy

**Keywords:** SR-A1, SR family, serine/arginine-rich proteins, splicing factors, RNA polymerase, pre-mRNA splicing, ovarian cancer, gene overexpression in cancer

## Abstract

By using the positional cloning gene approach, we were able to identify a novel gene encoding for a serine/arginine-rich protein, which appears to be the human homologue of the rat A1 gene. We named this new gene SR-A1. Members of the SR family of proteins have been shown to interact with the C-terminal domain (CTD) of the large subunit of RNA polymerase II and participate in pre-mRNA splicing. We have localized the SR-A1 gene between the known genes IRF3 and RRAS on chromosome 19q13.3. The novel gene spans 16.7 kb of genomic sequence and it is formed of 11 exons and 10 intervening introns. The SR-A1 protein is composed of 1312 amino acids, with a molecular mass of 139.3 kDa and a theoretical isoelectric point of 9.31. The SR-A1 protein contains an SR-rich domain as well as a CTD-binding domain present only in a subset of SR-proteins. Through interactions with the pre-mRNA and the CTD domain of the Polymerase II, SR proteins have been shown to regulate alternative splicing. The SR-A1 gene is expressed in all tissues tested, with highest levels found in fetal brain and fetal liver. Our data suggest that this gene is overexpressed in a subset of ovarian cancers which are clinically more aggressive. Studies with the steroid hormone receptor-positive breast and prostate carcinoma cell lines ZR-75-1, BT-474 and LNCaP, respectively, suggest that SR-A1 is constitutively expressed. Furthermore, the mRNA of the SR-A1 gene in these cell lines appears to increase by estrogens, androgens and glucocorticoids, and to a lesser extend by progestins. © 2001 Cancer Research Campaign http://www.bjcancer.com


*British Journal of Cancer* (2001) **85**(2), 190–198
© 2001 Cancer Research Campaign
doi: 10.1054/ bjoc.2001.1885, available online at http://www.idealibrary.com on IDEAL®

http://www.bjcancer.com

### Cloning of a gene (SR-A1), encoding for a new member of the human Ser/Arg-rich family of pre-mRNA splicing factors: overexpression in aggressive ovarian cancer

**A Scorilas[1,2]*, L Kyriakopoulou[1,2], D Katsaros[3] and EP Diamandis[1,2]**

[1]Department of Pathology and Laboratory Medicine, Mount Sinai Hospital, Toronto, Ontario M5G 1X5, Canada; [2]Department of Laboratory Medicine and Pathobiology, University of Toronto, Toronto, Ontario M5G 1L5, Canada; [3]Department of Gynecology, Gynecologic Oncology Unit, University of Turin, Turin, 10126, Italy

**Summary** By using the positional cloning gene approach, we were able to identify a novel gene encoding for a serine/arginine-rich protein, which appears to be the human homologue of the rat A1 gene. We named this new gene SR-A1. Members of the SR family of proteins have been shown to interact with the C-terminal domain (CTD) of the large subunit of RNA polymerase II and participate in pre-mRNA splicing. We have localized the SR-A1 gene between the known genes IRF3 and RRAS on chromosome 19q13.3. The novel gene spans 16.7 kb of genomic sequence and it is formed of 11 exons and 10 intervening introns. The SR-A1 protein is composed of 1312 amino acids, with a molecular mass of 139.3 kDa and a theoretical isoelectric point of 9.31. The SR-A1 protein contains an SR-rich domain as well as a CTD-binding domain present only in a subset of SR-proteins. Through interactions with the pre-mRNA and the CTD domain of the Polymerase II, SR proteins have been shown to regulate alternative splicing. The SR-A1 gene is expressed in all tissues tested, with highest levels found in fetal brain and fetal liver. Our data suggest that this gene is overexpressed in a subset of ovarian cancers which are clinically more aggressive. Studies with the steroid hormone receptor-positive breast and prostate carcinoma cell lines ZR-75-1, BT-474 and LNCaP, respectively, suggest that SR-A1 is constitutively expressed. Furthermore, the mRNA of the SR-A1 gene in these cell lines appears to increase by estrogens, androgens and glucocorticoids, and to a lesser extend by progestins. © 2001 Cancer Research Campaign http://www.bjcancer.com

**Keywords**: SR-A1; SR family; serine/arginine-rich proteins; splicing factors; RNA polymerase; pre-mRNA splicing; ovarian cancer; gene overexpression in cancer

Splicing of pre-mRNA requires multiple components that can form a stable spliceosome, and a number of factors that can stabilize the interaction of the spliceosome with the pre-mRNA molecule. Several nuclear ribonucleoprotein (RNP)-binding proteins have been identified, that are both essential for splicing and can influence splice-site selection (Spritz et al, 1987; Kohtz et al, 1994). Some of the best characterized factors belong to a large family of proteins that contain a serine/arginine-rich domain, thus named SR proteins (SR). SR proteins can complement splicing-inactive S100 fractions and alter splice-site selection in alternatively spliced pre-mRNAs (Zahler et al, 1993). Moreover, there is evidence that SR protein splicing factors are involved in cancer pathobiology through their effect on alternative processing decisions (Gobert et al, 1996; Stickeler et al, 1999).

In rat and yeast, the SR proteins have been characterized as part of a large complex which is directly associated with the C-terminal domain (CTD) of the large subunit of RNA polymerase II (CTD) (Yuryev et al, 1996), Detailed analyses of its interaction with the SR proteins suggests that the CTD domain, in complex with the SR proteins, may be involved in the early post-initiation events taking place during mRNA synthesis, including splicing. Yuryev et al employed the yeast 2-hybrid system, to identify proteins that interact with the CTD and cloned 4 distinct rat cDNAs that encode for high molecular mass SR proteins (Yuryev et al, 1996). The predicted rat SR proteins, namely rA1, rA4, rA8 and rA9 range in size from 120 to 140 kDa.

In humans, 9 distinct SR-encoding genes have been identified and characterized to date. These include the SF2/ASF and its alternatively spliced isoforms ASF-2 and ASF-3 (Ge et al, 1991), SC35 (Fu and Maniatis, 1992; Vellard et al, 1992), SRp20 (Kim et al, 1992), SRp30 (Screaton et al, 1995) SRp40 (Meier, 1996), SRp55 (Screaton et al, 1995), SRp75 (Zahler et al, 1993), 9G8 (Cavaloc et al, 1994) and SRrp129/CASP11 (Tanner et al, 1997). In addition, a set of high molecular weight proteins that cross-react with SR-specific monoclonal antibodies, has also been identified in human cell lines (Blencowe et al, 1994). However, only one gene encoding for a high molecular mass SR protein, SRrp129/CASP11, has been identified (Tanner et al, 1997). The SRrp129/CASP11 protein was shown to contain both an SR domain and CTD-binding domain. Similar to the rat SR proteins, the SRrp129/CASP11 protein was also implicated in RNA splicing (Tanner et al, 1997).

In this study we report the identification of a novel human gene, encoding for high molecular mass SR protein, distinct from the SRrp129/CASP11, that appears to be the human homologue of the rat A1 gene. The new gene was named SR-A1, and was identified by positional cloning. The human SR-A1 gene was mapped to

*Received 15 November 2000*
*Revised 3 April 2001*
*Accepted 5 April 2001*

*Correspondence to:* EP Diamandis

*Present Address: National Center for Scientific Research 'Demokritos', IPC, Athens 153 10 Greece. E-mail: Scorilas@netscape.net human chromosome 19 (19q13.3) and is localized between the known genes IRF3 and RRAS. The predicted protein is highly homologous to the rat A1 SR protein, and contains both signature domains present in the rat and human high molecular weight SR proteins, namely the CTD and SR. Levels of the SR-A1 transcripts were found to be increased in the breast and prostate carcinoma cell lines ZR-75-1, BT-474 and LNCaP, respectively, in response to hormone stimulation. In a subset of ovarian tumours tested, we found that higher expression of the SR-A1 gene is associated with clinically more aggressive disease.

### MATERIALS AND METHODS

### Gene identification and mapping

In our search for new SR-encoding genes involved in the process of pre-mRNA splicing, blast search was performed for different CTD-binding conserved motifs, using the TBLASTN program against the unfinished High Throughput Genomic Sequences database (htgs). A potential CTD-binding domain was identified in the BAC clone BC42053 sequenced by the Department of Energy's Joint Genome Institute. Genomic sequences from this clone and the particle overlapped BC 878087, were in the form of 167 contigs of different lengths. Restriction enzyme sites of the sequences were determined using the 'WebCutter 2' computer program. The chromosome 19 EcoR1 restriction map (Ashworth et al, 1995), information from BAC and PAC end sequences, and long PCR strategies and sequencing were used to construct a contiguous area of approximately 50 kb in length, of the genomic area of interest. The published sequences of the RRAS and IRF3 genes, were used to identify the relative positions of these genes on the contiguous map using the BLAST 2 algorithm (Altschul et al, 1997). Bioinformatic approaches were used to predict the presence of putative new genes within this genomic region (Murakami and Takagi, 1998; Scorilas et al, 2000, 2001; Yousef et al, 2000a, b, c, 2001).

### Expressed sequence tag (EST) searching

The predicted exons of the putative new gene were subjected to homology search using the BLASTN algorithm (Altschul et al, 1997) against the human EST database (dbEST). Clones with >95% identity were obtained from the I.M.A.G.E. consortium (Lennon et al, 1996) through Research Genetics Inc, Huntsville, AL and from the Institute for Genomic Research (TIGR), Rockville, MD. Clones were propagated and purified and then sequenced from both directions with an automated sequencer, using insert-flanking vector primers. All clones tested are shown in Table 1.

**Table 1** EST clones with >95% identity to exons of the SR-A1 gene

| GenBank accession no. | Tissue | I.M.A.GE. ID | Homologous exons |
|---|---|---|---|
| AW405880 | Testis | 3059116 | 2–7 |
| T85786 | Fetal liver | 112665 | 2–4 |
| AI806513 | Fetal lung | 2350047 | 4,5 |
| D30881 | Fetal lung | – | 7 |
| R88505 | Brain | 166268 | 9,10 |
| R90751 | Brain | 167214 | 9 |
| R88967 | Brain | 167004 | 9–11 |
| AI123319 | Fetal heart | 1689976 | 10 |
| AI688286 | Colon | 2325658 | 10,11 |
| AW473167 | Uterus | 2750431 | 10 |
| AA847768 | Colon tumour | 1419781 | 11 |
| AA568253 | Colon | 1057491 | 11 |
| AW298066* | Lung | 2732727 | 11 |
| AW296699* | Brain | 2730645 | 11 |

*Contains a Poly(A) tail.

### Protein homology searching

Putative exons of the newly identified gene were first translated to the corresponding amino acid sequences. BLAST homology searching for the proteins encoded by the exons were performed using the BLASTP algorithm and the GenBank databases (Altschul et al, 1997).

### Structure analysis of the SR-A1 protein

Multiple alignment was performed using the clustal X multiple alignment program (Aiyar, 2000). Phylogenetic studies were performed using the Phylip software package (Retief, 2000). Distance matrix analysis was performed using the 'Neighbor-Joining/UPGMA' program and parsimony analysis was done using the 'Protpars' program (Takezaki, 1998). Hydrophobicity study was performed using the Baylor College of Medicine search launcher program. Signal peptide and transmembrane regions prediction studies were performed using the SignalP (Nielsen et al, 1999) and Tmpred (Hofmann and Stoffel, 1992) software. Protein structure analysis was performed by SAPS program (Brendel et al, 1992). Sequence analysis tools were utilized to detect the presence of possible sites of post-translational modification on the SR-A1 protein. We used the analysis program PROSITE (Bairoch et al, 1997) and NetOGlyc 2.0 (Hansen et al, 1998) to detect N- and O-glycosylation, as well as the presence of kinase phosphorylation motifs.

### Cell lines and hormonal stimulation

The breast cancer cell lines ZR-75-1 and BT-474 as well as the prostate cancer cell line LNCaP were purchased from the American Type Culture Collection, Rockville, MD. Cells were cultured in RPMI media (Gibco BRL, Gaithersburg, MD) supplemented with glutamine (200 nmol $l^{-1}$), bovine insulin (10 mg $l^{-1}$), fetal bovine serum (10%), antibiotics and antimycotics, in plastic flasks, to near confluency. The cells were then aliquoted into 24-well tissue culture plates and cultured to 50% confluency. The culture media were changed to phenol red-free media containing 10% charcoal-stripped fetal bovine serum 24 hours prior to stimulation. Hormonal stimulation was performed by the addition of various steroid hormones, dissolved in 100% ethanol, to the culture media at a final concentration of $10^{-8}$M. Cells stimulated with 100% ethanol were included as negative controls. The cells were cultured for 24 hours before mRNA extraction.

### Tissue expression

Total RNA isolated from 28 different human tissues was purchased from Clontech, Palo Alto, CA. We prepared cDNA as described below, and used it for PCR reactions. Tissue cDNAs

© 2001 Cancer Research Campaign

**Table 2** Primers used for reverse transcription polymerase chain reaction (RT-PCR) analysis of the SR-A1 and 2 control genes

| Gene | Primer name | Sequence[a] | | | | | | | Length of PCR product |
|---|---|---|---|---|---|---|---|---|---|
| SR-A1 | F1 | CTC | ACC | CCA | TTA | GCT | GTC | CC | 695 |
| | R6 | GAT | CTC | TGT | CCC | CGA | TTC | GG | |
| | F6 | CCG | AAT | CGG | GGA | CAG | AGA | TC | 2500 |
| | R7 | GGA | CCA | GGA | AGT | CTC | CTC | CG | |
| | F7 | CGG | AGG | AGA | CTT | CCT | GGT | CC | 564 (F7/R9) |
| | R9 | CAT | CCC | CAG | ACT | TGA | GGG | TG | |
| | R11 | TCA | GGG | GTA | ATG | TTT | CAT | GA | 1302 (F7/R11) |
| Actin | ACTINS | ATC | TGG | CAC | CAC | ACC | TTC | TA | |
| | ACTINAS | CGT | CAT | ACT | CCT | GCT | TGC | TG | 838 |

[a]All nucleotide sequences are given in the 5′ → 3′ orientation.

were amplified using the primers F7 and R9, described in Table 2. PCR products were gel-purified and sequenced.

### Ovarian cancer and non-cancer tissues

Included in this study were specimens from 40 cancer and 5 non-cancer ovarian tissues. Patients were undergoing surgical treatment for primary ovarian carcinoma at the Department of Gynecology and Gynecologic Oncology Unit at the University of Turin, Turin, Italy, during the period from January 1996 to December 1998. This study had been approved by the Ethics and Research Committee at the institutional Review Board of the University of Turin. Normal ovaries were removed from patients who underwent surgery for benign non-ovarian pathology. Patient age ranged from 33–78 years with a median of 60. All patients had a histologically confirmed diagnosis and received no treatment before surgery. Classification followed the World Health Organization criteria (Serov and Sorbin, 1973). Clinical staging was performed according to the International Federation of Gynecology and Obstetrics (FIGO) staging system (Pettersson, 1994). Histologic grading was performed according to the criteria of Day et al; highly differentiated tumours were grade 1 and undifferentiated tumours grade 3 (Day et al, 1975). Ovarian tissue had been frozen in liquid nitrogen immediately after surgery. We prepared cDNA as described below and used it for PCR reactions with the primers F7 and R9. Tissue cDNAs were amplified at a 1:20 dilution (Kim et al, 2001).

### Reverse transcription polymerase chain reaction (RT-PCR)

Total RNA was extracted from the tissues or cell lines using Trizol ™ reagent (Gibco BRL) following the manufacturer's instructions. RNA concentration was determined spectrophotometrically. 2 $\mu$g of total RNA was reverse transcribed into first strand cDNA using the Superscript™ preamplification system (Gibco BRL, Gaithersburg MD). The final volume was 20 $\mu$l. Based on the combined information obtained from the predicted genomic structure of the new gene and the EST sequences, 2 gene-specific primers were designed (F7 and R9; Table 2) and PCR was carried out in a reaction mixture containing 1 $\mu$l of cDNA, 10 mM Tris-HCl (pH 8.3), 50 mM KCl, 1.5 mM $MgCl_2$, 200 μM dNTPs (deoxynucleoside triphosphates), 150 ng of primers and 2.5 units of AmpliTaq Gold DNA polymerase (Roche Molecular Systems, Branchburg, NJ, USA) on a Perkin-Elmer 9600 thermal cycler. The cycling conditions were a denaturation step at 95°C for 15 minutes, followed by 42 cycles of 94°C for 30 s, 64°C for 1 min and a final extension at 64°C for 10 min. Equal amounts of PCR products were electrophoresed on 2% agarose gels and visualized by ethidium bromide staining. The primers for RT-PCR spanned at least 2 exons to avoid contamination by genomic DNA.

### Cloning and sequencing of the PCR products

To verify the identity of the PCR products, they were cloned into the pCR 2.1-TOPO vector (Invitrogen, Carlsbad, CA, USA) according to the manufacturer's instructions. The inserts were sequenced from both directions using vector-specific primers, by an automated DNA sequencer.

### Statistical analysis

Statistical analysis was performed with SAS software (SAS Institute, Cary, NC). Ovarian tumour SR-A1 mRNA levels were qualitatively classified into 2 categories (SR-A1 – low and SR-A1 – high groups). All samples were evaluated for SR-A1 expression by 2 observers who were unaware of patient outcome. Associations between SR-A1 status and other qualitative variables were analysed using the Fisher's exact test. Because of the small number of cases, tumour grade was categorized into 2 groups: I and II–III. The stage of the disease was also categorized into 2 groups: I–II and III–IV. The analysis of differences in SR-A1 status and age or residual tumour size was performed with the nonparametric Mann–Whitney U test.

### RESULTS

### Cloning of the SR-A1 gene

Analysis of the constructed contiguous genomic sequences in the region of chromosome 19q13.3 predicted a putative new gene formed of 11 exons. To verify the existence of this gene, the putative exons were subjected to sequence homology search against the human expressed sequence tag database (dbEST), and 14 EST clones with > 95% identity were found (Table 1). Some of these clones were obtained and inserts were sequenced from both directions. Sequences were compared with the computer-predicted structure and final selection of the intron/exon splice sites was

© 2001 Cancer Research Campaign done according to the EST sequences. The ESTs matched well with most of the predicted exons of the gene and 2 of them possessed a poly(A) tail, confirming the 3′ end of the mRNA (Table 1). Moreover, homology search of the exons against the GenBank database revealed a rat cDNA with 84% identity. This cDNA encodes for the rat A1 protein, an SR-like protein, and matches with exons 3–11 our novel human gene (see below).

To verify the accuracy of the cDNA sequence, PCR reactions were performed using 3 pairs of primers, specific for various exons of the gene (F1/R6 extending from exons 1–6; F6/R7 for exons 6–7 and F7/R11 for exons 7–11; see Table 2) with cDNA isolated from different human tissues as putative templates. The PCR bands were cloned and sequenced to further define and confirm the exon/intron boundaries. The genomic and cDNA sequence of the new gene is now deposited in GenBank (accession # AF254411).

### Mapping and chromosomal localization of the SR-A1 gene

Alignment of the SR-A1 gene sequence and the sequences of other known genes localized within the same area of the chromosome 19q13.3 enabled us to precisely localize these genes and determine the direction of the transcription, as shown in Figure 1. The SR-A1 gene maps between 2 known genes, namely IRF3 and RRAS and is transcribed in the opposite direction. The distances between SR-A1 and IRF3 and RRAS were calculated to be 921 bp and 1821 bp, respectively. We did not predict any other genes between IRF3 or RRAS.

### Characterization of the genomic structure of the SR-A1 gene and its protein product

The SR-A1 gene, as diagrammatically presented in Figure 2, is formed of 11 exons and 10 intervening introns, spanning an area of 16 730 bp of genomic sequence on chromosome 19q13.3. The lengths of the exons are 323, 114, 58, 95, 101, 116, 2838, 83, 219, 129, and 442 bp, respectively. All the intron/exon splice sites and their flanking sequences are closely related to the consensus splice sites (-m**GT**AAGT...C**AG**m-, where m is any base) (Iida, 1990).

The protein coding region of the novel gene consists of 3939 nucleotides (including the stop codon), producing a 1312 amino acid protein, with a predicted molecular mass of 139.3 kDa, excluding any post-translational modifications, and a theoretical isoelectric point of 9.31. The translation initiation codon (ATG) at position 7 of the second exon, was chosen because: (1) The flanking sequence of this codon (ACC**ATG**G) matches closely with the Kozak consensus sequence (GCC A/G CCATGG) for initiation of translation (Kozak, 1991); (2) No other start codon located within the first 4 exons has the capacity to generate a functional, in-frame mRNA with the remaining exons; (3) Using this initiation codon, the translated product is similar in length and has a high degree of homology with other similar proteins (see below). Thus, we conclude that the first exon is untranslated. The 3′ terminus of the SR-A1 gene was verified by the presence of a poly (A) tail in two EST sequences (Table 1). As it is evident from Figure 2, this gene possesses a 5′ untranslated region of at least 329 nucleotides, as well as a 3′ untranslated region of 250 nucleotides.

To more extensively characterize the predicted structural motifs of the SR-A1 protein, the SR-A1 protein was aligned with other members of the SR multi-gene family (Figure 3). SR-A1 is found to have 78% amino acid identity and 84% similarity with the rat A1 protein suggesting that the newly identified SR-A1 gene is the human homologue of the rat A1 gene, reported by Yuryev et al (Yuryev et al, 1996). The SR-A1 protein showed 24% identity with the rA9 gene product and 19% identity with the rA4 and rA8. The SR-A1 protein showed 22% amino acid identity and 33% similarity with the SRrp129 protein and to a lesser extend (< 20%) with the low molecular mass SR proteins. As shown in Figures 2 and 3, common SR domains present in the rat A1 and the other SR proteins were also identified in the SR-A1 predicted protein. The CTD-binding domain of rat A1, rat A9 and SRrp129 protein was also present in the SR-A1 protein. Also, two areas with negatively charged polyglutamic acid (E) stretches which are present in rat A1 but not in other known SR proteins, were identified (Figures 2, 3). In addition, we identified the presence of an Arg/Asp-rich motif (RD) in the SR-A1 protein. This motif is also present in a number of other RNA binding proteins such as the U1-70 K (Spritz et al, 1987), the RD RNA-binding protein (Surowy et al, 1990) and the 68 kDa human pre-mRNA cleavage factor $I_m$ (Ruegsegger et al, 1998). In addition to the above-described motifs, this protein is also proline-rich and contains a large number of PS or SP dinucleotides ($n$ = 39) and a large number of PAP trinucleotides ($n$ = 16). Overall, the percentage of various amino acids represented in this protein are S, 13.6%; P, 13.3%; R, 9.8%, A, 9.3%; E, 8.5% and other amino acids are less than 7%. Other long stretches of single amino acids (>5) are shown in Figure 2.

To predict the phylogenetic relatedness of the SR-A1 with other SR proteins, the amino acid sequences of these genes were aligned using the ‘Clustal X’ multiple alignment program and a distance matrix tree was predicted using the Neighbour-joining/UPGMA method. Figure 4 shows that the SR-A1 is grouped with rat A1 protein, whereas the other known SR-proteins were separated in other groups.

Examination of the hydrophobicity profile of the SR-A1 protein did not reveal regions with long stretches of hydrophobic residues (Figure 5). This is consistent with findings from a signal sequence prediction program (Nielsen et al, 1999) which did not predict a signal sequence. SR-A1 protein is predicted to be a nuclear protein with no transmembrane region, in agreement with the rat homologue protein. Through the use of sequence analysis tools, we were able to identify various putative post-translational modification sites (Table 3). There are numerous potential sites for either O- or N-glycosylation. Furthermore, several possible sites of phosphorylation have been identified for cAMP-dependent protein kinase, protein kinase C, and casein kinase 2.

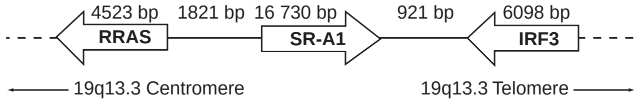

**Figure 1** An approximate 35 kb region of contiguous genomic sequence around chromosome 19q13.3. Genes are represented by horizontal arrows denoting the direction of transcription. Gene lengths and distances between genes are mentioned in base pairs (bp). Figure is not drawn to scale. IRF3, interferon regulatory factor 3

### Tissue expression profile of the SR-A1 gene

RT-PCR was performed on a panel of total RNA preparations extracted from various tissues. RT-PCR for actin was used as a positive control. Positive ESTs for SR-A1 were also used as controls. The tissue expression of SR-A1 is summarized in Figure 6.

© 2001 Cancer Research Campaign 

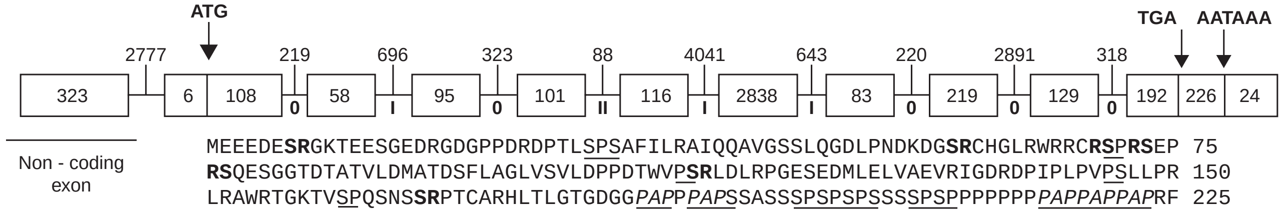

```
MEEEDESRGKTEESGEDRGDGPPDRDPTLSPSAFILRAIQQAVGSSLQGDLPNDKDGSRCHGLRWRRCRSPRSEP 75
RSQESGGTDTATVLDMATDSFLAGLVSVLDPPDTWVPSRLDLRPGESEDMLELVAEVRIGDRDPIPLPVPSLLPR 150
LRAWRTGKTVSPQSNSSRPTCARHLTLGTGDGGPAPPPAPSSASSSPSPSPSSSSPSPPPPPPPPAPPAPPAPRF 225
DIYDPFHPTDEAYSPPPAPEQKYDPFEPTGSNPSSSAGTPSPEEEEEEEEEEEEEEEDEEEEEGLSQSISRISET 300
LAGIYDDNSLSQDFPGDESPRPDAQPTQPTPAPGTPPQVDSTRADGAMRRRVFVVGTEAEACREGKVSVEVVTAG 375
GAALPPPLLPPGDSEIEEGEIVQPEEEPRLALSLFRPGGRAARPTPAASATPTAQPLPQPPAPRAPEGDDFLSLH 450
AESDGEGALQVDLGEPAPAPPAADSRWGGLDLRRKILTQRRERYRQRSPSPAPAPAPAAAAGPPTRKKSRRERKR 525
SGEAKEAASSSSGTQPAPPAPASPWDSKKHRSRDRKPGSHASSSARRRSRSRSRSRSTRRRSRSTDRRRGGSRRS 600
RSREKRRRRRRSASPPPATSSSSSSRRERHRGKHRDGGGSKKKKKRSRSRGEKRSGDGSEKAPAPAPPPSGSTSC 675
GDRDSRRRGAVPPSIQDLTDHDLFAIKRTITVGRLDKSDPRGPSPAPASSPKREVLYDSEGLSGEERGGKSSQKD 750
RRRSGAASSSSSSREKGSRRKALDGGDRDRDRDRDRDRDRSSKKARPPKESAPSSGPPPKPPVSSGSGSSSSSSS 825
CSSRKVKLQSKVAVLIREGVSSTTPAKDAASAGLGSIGVKFSRDRESRSPFLKPDERAPTEMAKAAPGSTKPKKT 900
KVKAKAGAKKTKGTKGKTKPSKTRKKVRSGGGSGGSGGQVSLKKSKADSCSQAAGTKGAEETSWSGEERAAKVPS 975
TPPPKAAPPPPALTPDSQTVDSSCKTPEVSFLPEEATEEAGVRGGAEEEEEEEEEEEEEEEEEEQQPATTTATST 1050
AAAAPSTAPSAGSTAGDSGAEDGPASRVSQLPTLPPPMPWNLPAGVDCTTSGVLALTALLFKMEEANLASRAKAQ 1125
ELIQATNQILSHRKPPSSLGMTPAPVPTSLGLPPGPSSYLLPGSLPLGGCGSTPPTPTGLAATSDKREGSSSSEG 1200
RGDTDKYLKKLHTQERAVEEVKLAIKPYYQKKDITKEEYKDILRKAVHKICHSKSGEINPVKVSNLVRAYVQRYR 1275
YFRKHGRKPGDPPGPPRPPKEPGPPDKGGPGLPLPPL 1312
```

**Figure 2** Schematic representation of the SR-A1 gene and the amino acid sequence of the predicted SR-A1 protein. Exons are shown as boxes and introns as connecting lines. Arrows show the positions of the start codon, stop codon and polyadenylation signal. Roman numerals indicate intron phases. The intron phase refers to the location of the intron within the codon; I denotes that the intron occurs after the first nucleotide of the codon, II that the intron occurs after the second nucleotide, and 0 that the intron occurs between codons. Numbers inside boxes indicate exon lengths and the vertical connecting lines show the intron lengths (in bp). Figure is not drawn to scale. RS or SR dinucleotides are shown in bold and the CTD-binding domain of the SR-A1 protein is boxed. An Arg/Asp rich motif (RD) is double-underlined. There are 2 areas with polyglutamic acid (E) stretches which are shown in italics. Other putative motifs (PS, SP, PAP) and repeated aminoacids, (≥ 5) are underlined. The full genomic organization of the gene is reported in GenBank (accession # AF254411)

**Table 3** Putative post-translational modification sites in the novel SR-A1 gene

| Modification | Residue | Position[a] |
|---|---|---|
| | Thr | 93, 264, 327, 330, 335, 420, 426, 428, 539, 619, 976, 994, 1044–48, 1050, 1057, 1083. |
| O-glycosylation | Ser | 191, 194–96, 198, 200, 202–205, 207, 239, 259, 260, 261, 424, 498, 500, 536, 537, 620, 670, 719, 804, 805, 814, 815, 817, 819, 1049, 1056, 1177. |
| N-glycosylation | Asn | 165. |
| cAMP-dependent protein kinase phosphorylation | Ser | 516, 523, 571, 584, 609, 751. |
| Protein kinase C phosphorylation | Ser | 70, 166, 319, 341, 519, 552, 569, 582, 597, 624, 625, 640, 680, 762, 768, 792, 827. |
| | Thr | 156, 488, 515, 923, 583, 590. |
| Casein kinase 2 phosphorylation | Ser | 14, 102, 113, 266, 389, 453, 519, 548, 625, 649, 674, 689, 738, 747, 762, 965. |
| | Thr | 87, 342, 357, 694. |

[a]The residues are numbered according to the sequence shown in Figure 1.

**Table 4** Relationship between SR-A1 expression (low or high) status and other clinicopathological variables in ovarian cancer patients

| Variable | Patients | No. of patients (%) SR-A1 low | SR-A1 high | *P* value[a] |
|---|---|---|---|---|
| Grade[b] | | | | |
| I | 5 | 4 (75.0%) | 1 (25.0%) | < 0.001 |
| II-III | 33 | 3 (9.1%) | 30 (90.9%) | |
| Stage[c] | | | | |
| I-II | 11 | 6 (54.5%) | 5 (45.5%) | 0.007 |
| III-IV | 29 | 3 (13.3%) | 26 (89.7%) | |
| Debulking[d] | | | | |
| OD | 17 | 7 (41.2%) | 10 (58.8%) | 0.023 |
| SD | 23 | 2 (8.7%) | 21 (91.3%) | |

[a]Fisher's exact test. [b]B, borderline; I, well differentiated; II, moderately differentiated; III, poorly differentiated. [c]International Federation of Gynaecology and Obstetrics (FIGO) staging system. [d]OD, optimal (0–1 cm); SD, suboptimal (> 1 cm).

The novel gene is highly expressed in many tissues. Highest levels of SR-A1 transcripts were detected in the fetal brain and fetal liver and the lowest in the salivary gland, heart, skin and ovary but virtually every tissue tested was positive. Cloning and sequencing of the PCR products ensured specificity.

### Expression of SR-A1 in cancer cell lines

We used the steroid hormone receptor-positive breast and prostate carcinoma cell lines ZR-75-1, BT-474 and LNCaP, respectively, as model systems to evaluate whether SR-A1 is expressed in these cells. As shown in Figure 7, we could detect expression of SR-A1 in all 3 cell lines and noticed that the expression was affected by steroid hormone treatment. Specifically, the mRNA of the SR-A1 gene in ZR-75-1 and LNCaP cell lines appears to increase by oestrogens, androgens and glucocorticoids, and to a lesser extend by progestins. However, since no promoter characterization studies have been performed, we do not know if the effect of steroid hormones on SR-A1 transcription is direct or indirect in nature.

 *© 2001 Cancer Research Campaign*

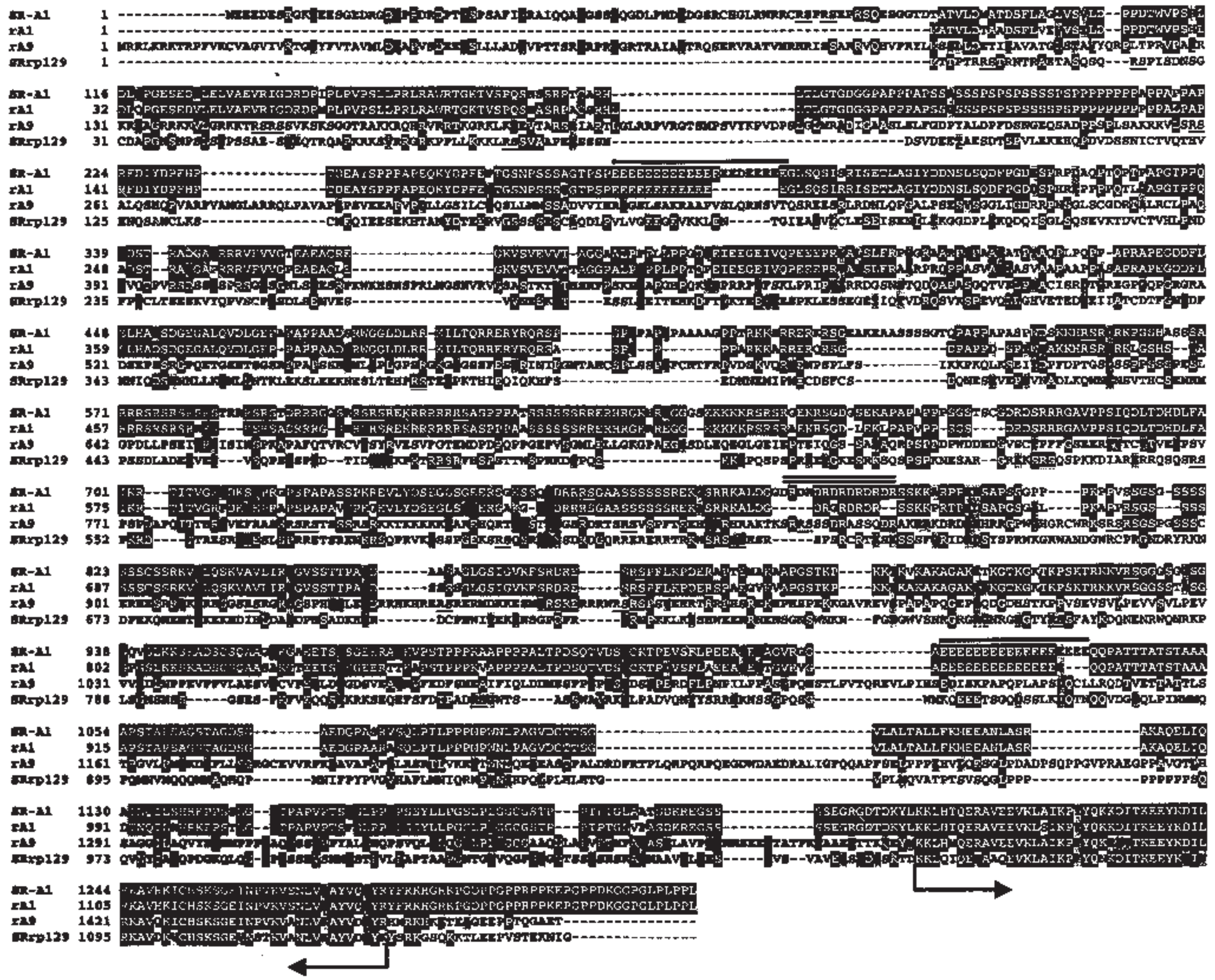

**Figure 3** Alignment of the deduced amino acid sequence of SR-A1 with members of the SR gene family (rat A1, rat A9 and human SRrp129). Dashes represent gaps to bring the sequences to better alignment. Conserved areas are highlighted in grey. The arrows mark start and end of the CTD-binding domain. 2 areas with polyglutamic acid stretches are marked with a solid line on top. The RD motif is marked with a solid double line on top. Maximum homology between all 4 proteins is seen in the area of the CTD domain

### SR-A1 expression in ovarian cancer

Expression of the SR-A1 gene, at the mRNA level, was examined in 40 ovarian tumours and 5 non-cancerous ovarian tissue specimens, using RT-PCR. Actin was a positive control. Examples of SR-A1 gene expression in normal ovarian tissue, and in tumours with low or hight expression are shown in Figure 8. The distributions of SR-A1 qualitative expression status (high or low) between subgroups of patients differing by tumour grade, disease stage and success of debulking were examined by the Fisher's exact test (Table 4). SR-A1-high expression was found more frequently in grade II–III, stage III–IV and suboptimal debulking patients ($P < 0.001$, $P = 0.007$ and $P = 0.023$, respectively). Differences in residual tumour size between the 2 SR-A1 status patients groups were found to be statistically significant by the Mann–Whitney test ($P = 0.006$). Median values of the residual tumour size were 0 and 3 for SR-A1-low and SR-A1-high tumours, respectively. Although only 2 borderline tumours were available, it is worth mentioning that the SR-A1 gene was not expressed in the above tumours. Statistically significant association between SR-A1 status and patient age was not observed.

### DISCUSSION

We have identified a human gene encoding for a new member of the SR protein family. Using positional candidate gene analysis we identified a gene spanning an area of 16.7 kb of genomic sequence and we named it SR-A1, based on homologies with other members of the SR protein family. EST analysis was used to delineate the genomic organization of the gene and predict the putative mRNA coding region. RT-PCR and sequencing were used verify the splice junctions. The SR-A1 gene is formed of 11 coding exons and 10 intervening introns. Present at the 3′-terminus of 2 ESTs was a poly-A tail, while the 5′-terminus of the mRNA coding region, contained a well-conserved Kozak sequence (Kozak, 1991). The new gene has 84% nucleotide identity to the rat A1 gene (rA1), which is a member of the SR protein family. The human SR-A1 protein has highly-conserved SR and CTD-binding domains as

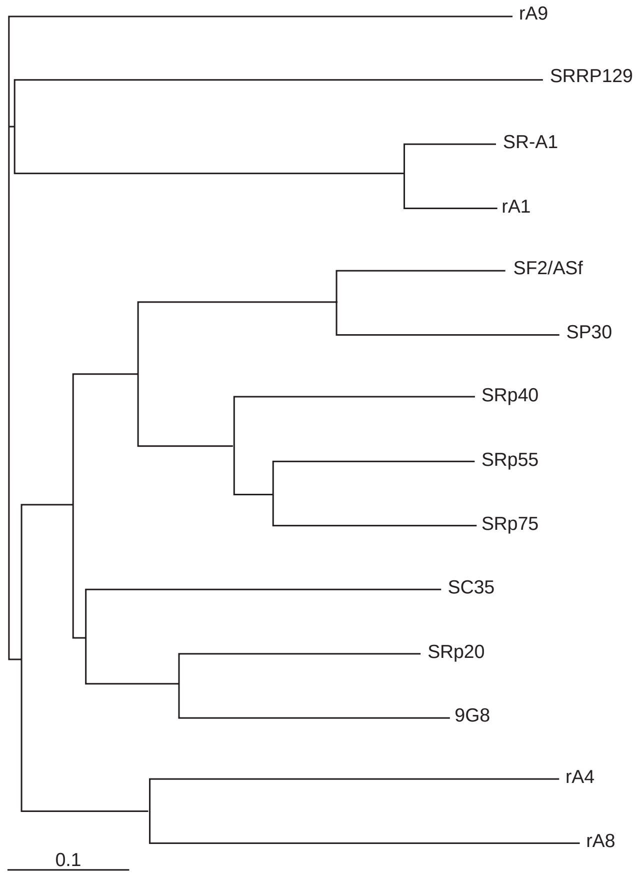

**Figure 4** Dendrogram of the predicted phylogenetic tree for the known SR proteins. Neighbour-joining/UPGMA method was used to align SR-A1 with other members of the SR gene family. The tree aligned the SR-A1 protein in one group with the rat A1 gene. Other SR proteins were aligned in different groups well as the Arg/Asp rich motif and 2 stretches of polyglutamic acid areas, also found in rat A1. For these reasons, we consider our new gene as the human homologue of rA1 (Yuryev et al, 1996).

The SR protein family is very conserved, with molecular weights of 20, 30, 40, 55 and 75 kDa, described in a number of organisms (Zahler et al, 1993; Screaton et al, 1995). Human SR proteins are encoded by nine distinct SR genes, and their sizes range from 20 to 129 kDa. In addition, a number of alternatively spliced isoforms have been reported (Screaton et al, 1995). A group of high molecular weight SR proteins has also been identified, by their reactivity with the monoclonal antibody mAb104, that recognizes an epitope of alternating phosphoserine and arginine residues, and also by the fact that they can be readily precipitated by $Mg^{2+}$, a feature shared by all SR proteins (Blencowe et al, 1994). To our knowledge, no corresponding genes or cDNAs have as yet been identified for these proteins.

The predicted protein coding region of the SR-A1 gene is 3,939 bp, encoding for a 1312-amino acid polypeptide with a predicted molecular weight of 139.3 kDa. This molecular weight is similar to the one reported for the rA1 protein (Yuryev et al, 1996) and the human SRrp129/CASP11 protein (Tanner et al, 1997). Furthermore, the SR-A1 protein has 78% and 22% identity with the rat A1 and human SRrp129/CASP11 protein, respectively. The SR domains of the rat A1 are also present in the human SR-A1 protein. Thus, the SR-A1 protein appears to be a novel member of the human SR family.

Several lines of evidence suggest that the SR proteins play a role in splicing (Zahler et al, 1993; Blencowe et al, 1994; Yuryev et al, 1996), perhaps by linking the synthesis of mRNA with the processing mechanism, specifically by regulating alternative splicing (McKeown et al, 1987; Amrein et al, 1988; Goralski et al, 1989). Furthermore, an Arg/Asp (RD)-rich motif present in the predicted SR-A1 protein was also found to be present in the RNA-binding proteins U1-70 K (Spritz et al, 1987), the RD RNA-binding protein (Surowy et al, 1990) and the 68 kDa human pre-mRNA cleavage factor $I_m$ (Ruegsegger et al, 1998). The latter, has been implicated in processing of the 3′ end and in the regulation of the polyadenylation process (Takagaki et al, 1989, 1992). The same RD motif has also been identified in the rat SR proteins rA1, rA4 and rA8, but not the rA9 (Yuryev et al, 1996).

Members of the SR family have been shown to exhibit overlapping functions but can modulate alternative splicing of specific pre-mRNAs (Wang et al, 1998). It has also been postulated that the SR proteins participate in tumorigenesis by regulating alternative splicing of several mRNAs such as the CD44 pre-mRNA splicing in ovarian as well as mammary cancer (Cannistra et al, 1993; Stickeler et al, 1999).

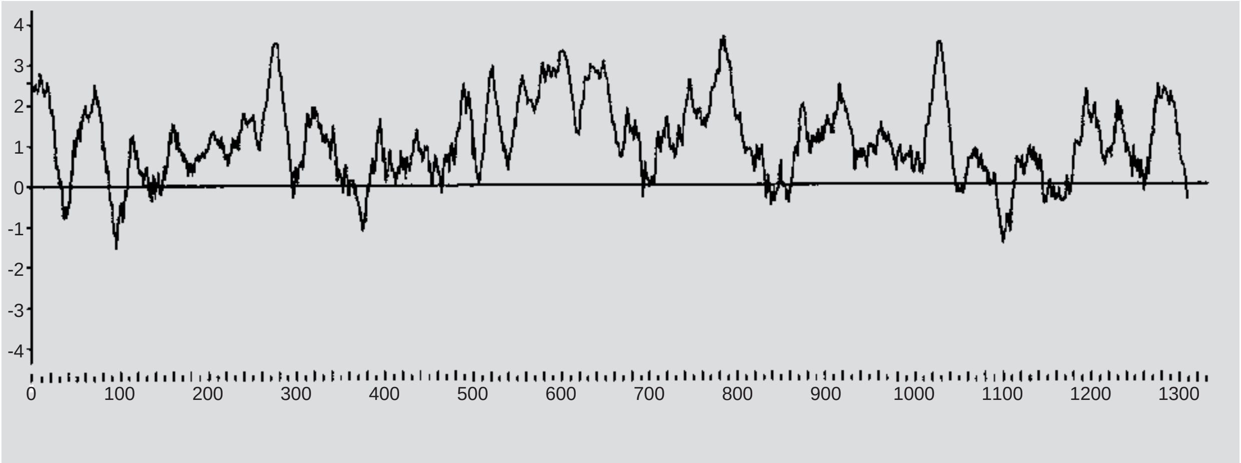

**Figure 5** Plot of hydrophobicity and hydrophilicity of SR-A1 protein. For discussion see text

 *© 2001 Cancer Research Campaign*

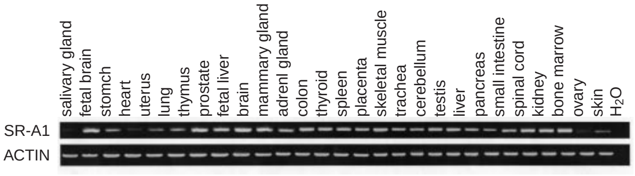

**Figure 6** Tissue expression of SR-A1 gene as determined by RT-PCR. Actin is a control gene. SR-A1 is widely expressed in all tested tissues

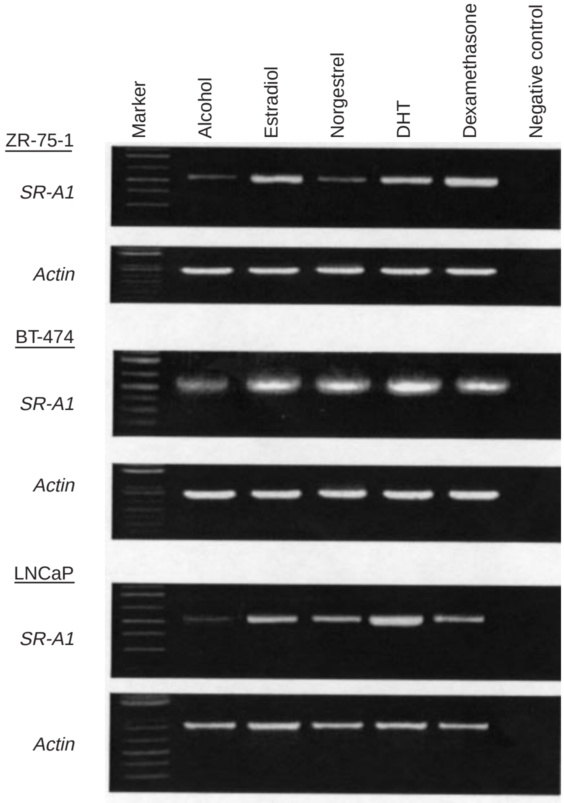

**Figure 7** Representative RT-PCR reactions on mRNA from breast and prostate cancer cell lines (ZR-75–1, BT-474 and LNCaP, respectively) stimulated with various steroid hormones. The same cDNAs were used for actin gene amplification (control). Steroids were at $10^{-8}$ M final concentration. For discussion, see text

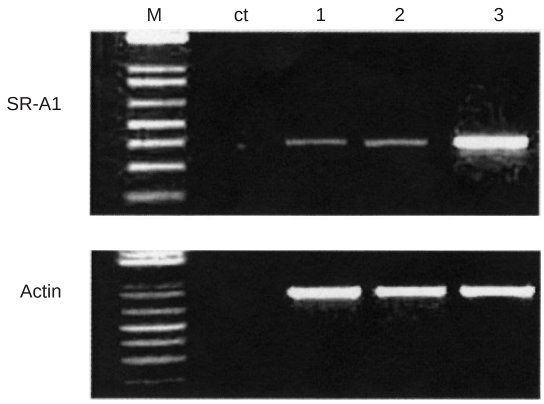

**Figure 8** Expression of the SR-A1 gene in the ovary. M, DNA molecular weight marker; ct, negative control; 1, normal ovary; 2, ovarian tumour with low expression of the SR-A1 gene; 3, ovarian tumour with high expression of the SR-A1 gene. Actin is a control gene. For more comments see text

The human SR-A1 gene is located on chromosome 19q13.3, between the RRAS and IRF3 genes (Figure 1). The connection of the RAS genes to human malignancy is well-known (Webb et al, 1998). IRF3 is a member of a growing family of transcription factors. It is becoming increasingly apparent that IRF3 plays a pivotal role in the cell's response to viral infection. Phosphorylation and activation of IRF3 in response to stimuli other than viral infection may result in transcription of genes, in addition to the early inflammatory genes (Au et al, 1995; Lowther et al, 1999). Activation of IRF3 by growth factors can lead to a negative feedback control of cell growth (Au et al, 1995).

The human SR-A1 gene was shown to be expressed widely in all normal tissues tested. However, the level of the SR-A1 transcripts seem to vary between tissues. The highest level of SR-A1 transcripts was detected in the fetal brain and fetal liver and the lowest in salivary gland, skin, uterus and ovary (Figure 6).

Several ovarian tumour extracts were examined with respect to SR-A1 expression. SR-A1 transcripts were shown to be differentially regulated in ovarian tumors. The pattern of exprression appears to correlate significantly with tumour grade, disease stage and success of debulking (Table 4). The differential expression of SR-A1 in ovarian tumours suggests that the SR-A1 protein may participate in alternative splicing processes during progression of ovarian tumours. Our data suggest that SR-A1 overexpression is associated with clinically more aggressive ovarian tumours.

In the mammary and prostate gland SR-A1 gene transcripts are constitutively present at relatively high levels (Figure 6). The level of the SR-A1 mRNA appears to increase in cell lines treated with various steroid hormons (Figure 7). However, the mechanism of this event needs further investigation.

In conclusion, we cloned a gene that encodes for a novel SR-protein, which appears to be the human homologue of the rat A1 gene cloned by Yuryev et al (1996). This gene is localized between the 2 known genes; IRF3 and RRAS on chromosome 19q13.3. We report that this gene is overexpressed in a subset of clinically more aggressive ovarian cancers and speculate that SR-A1 may be involved in the pathogenesis and/or progression of ovarian and other cancers.

### REFERENCES

Aiyar A (2000) The use of CLUSTAL W and CLUSTAL X for multiple sequence alignment. *Methods Mol Biol* **132**: 221–241

Altschul SF, Madden TL, Schaffer AA, Zhang J, Zhang Z, Miller W and Lipman DJ (1997) Gapped BLAST and PSI-BLAST: a new generation of protein database search programs. *Nucleic Acids Res* **25**: 3389–3402

Amrein H, Gorman M and Nothiger R (1988) The sex-determining gene tra-2 of Drosophila encodes a putative RNA binding protein [published erratum appears in Cell 1989 Jul 28;58(2): following 419]. *Cell* **55**: 1025–1035

Ashworth LK, Batzer MA, Brandriff B, Branscomb E, de Jong P, Garcia E, Garnes JA, Gordon LA, Lamerdin JE, Lennon G, et al (1995). An integrated metric physical map of human chromosome 19. *Nat Genet* **11**: 422–427

Au WC, Moore PA, Lowther W, Juang YT and Pitha PM (1995) Identification of a member of the interferon regulatory factor family that binds to the interferon-stimulated response element and activates expression of interferon-induced genes. *Proc Natl Acad Sci USA* **92**: 11657–11661

Bairoch A, Bucher P and Hofmann K (1997) The PROSITE database, its status in 1997. *Nucleic Acids Res* **25**: 217–221

Blencowe BJ, Nickerson JA, Issner R, Penman S and Sharp PA (1994) Association of nuclear matrix antigens with exon-containing splicing complexes. *J Cell Biol* **127**: 593–607

Brendel V, Bucher P, Nourbakhsh IR, Blaisdell BE and Karlin S (1992) Methods and algorithms for statistical analysis of protein sequences. *Proc Natl Acad Sci USA* **89**: 2002–2006

*© 2001 Cancer Research Campaign* 

Cannistra SA, Kansas GS, Niloff J, DeFranzo B, Kim Y and Ottensmeier C (1993) Binding of ovarian cancer cells to peritoneal mesothelium in vitro is partly mediated by CD44H. *Cancer Res* **53**: 3830–3838
Cavaloc Y, Popielarz M, Fuchs JP, Gattoni R and Stevenin J (1994) Characterization and cloning of the human splicing factor 9G8: a novel 35 kDa factor of the serine/arginine protein family. *Embo J* **13**: 2639–2649
Day TG, Jr Gallager HS and Rutledge FN (1975) Epithelial carcinoma of the ovary: prognostic importance of histologic grade. *Natl Cancer Inst Monogr* **42**: 15–21
Fu XD and Maniatis T (1992) Isolation of a complementary DNA that encodes the mammalian splicing factor SC35. *Science* **256**: 535–538
Ge H, Zuo P and Manley JL (1991) Primary structure of the human splicing factor ASF reveals similarities with Drosophila regulators. *Cell* **66**: 373–382
Gobert C, Bracco L, Rossi F, Olivier M, Tazi J, Lavelle F, Larsen AK and Riou JF (1996) Modulation of DNA topoisomerase I activity by p53. *Biochemistry* **35**: 5778–5786
Goralski TJ, Edstrom JE and Baker BS (1989) The sex determination locus transformer-2 of Drosophila encodes a polypeptide with similarity to RNA binding proteins. *Cell* **56**: 1011–1018
Hansen JE, Lund O, Tolstrup N, Gooley AA, Williams KL and Brunak S (1998) NetOglyc: prediction of mucin type of O-glycosylation sites based on sequence context and surface accessibility. *Glycoconj J* **15**: 115–130
Hoffmann K and Stoffel W (1992) PROFILEGRAPH: an interactive graphical tool for protein sequence analysis. *Comput Appl Biosci* **8**: 331–337
Iida Y (1990) Quantification analysis of 5′-splice signal sequences in mRNA precursors. Mutations in 5′-splice signal sequence of human beta-globin gene and beta-thalassemia. *J Theor Biol* **145**: 523–533
Kim H, Scorilas A, Katsaros D, Yousef GM, Massobrio M, Fracchioli S, Piccinno R, Gordini G and Diamandis EP (2001) Human kallikrein gene 5 (KLK5) expression is an indicator of poor prognosis in ovarian cancer. *Br J Cancer* **84**: 643–650
Kim YJ, Zuo P, Manley JL and Baker BS (1992) The Drosophila RNA-binding protein RBPI is localized to transcriptionally active sites of chromosomes and shows a functional similarity to human splicing factor ASF/SF2. *Genes Dev* **6**: 2569–2579
Kohtz JD, Jamison SF, Will CL, Zuo P, Luhrmann R, Garcia-Blanco MA and Manley JL (1994) Protein-protein interactions and 5′-splice-site recognition in mammalian mRNA precursors. *Nature* **368**: 119–124
Kozak M (1991) An analysis of vertebrate mRNA sequences: intimations of translational control. *J Cell Biol* **115**: 887–903
Lennon G, Auffray C, Polymeropoulos M and Soares MB (1996) The I.M.A.G.E. Consortium: an integrated molecular analysis of genomes and their expression. *Genomics* **33**: 151–152
Lowther WJ, Moore PA, Carter KC and Pitha PM (1999) Cloning and functional analysis of the human IRF-3 promoter. *DNA Cell Biol* **18**: 685–692
McKeown M, Belote JM and Baker BS (1987) A molecular analysis of transformer, a gene in Drosophila melanogaster that controls female sexual differentiation. *Cell* **48**: 489–499
Meier UT (1996) Comparison of the rat nucleolar protein nopp 140 with its yeast homolog SRP40. Differential phosphorylation in vertebrates and yeast. *J Biol Chem* **271**: 19376–19384
Murakami K and Takagi T (1998) Gene recognition by combination of several gene-finding programs. *Bioinformatics* **14**: 665–675
Nielsen H, Brunak S and von Heijne G (1999) Machine learning approaches for the prediction of signal peptides and other protein sorting signals. *Protein Eng* **12**: 3–9
Pettersson F (1994). Annual report on the treatment in gynecological cancer Stocklolm: International Federation of Gynecology and Obstetrics Vol. 22. FIGO
Retief JD (2000) Phylogenetic analysis using PHYLIP. *Methods Mol Biol* **132**: 243–258
Ruegsegger U, Blank D and Keller W (1998) Human pre-mRNA cleavage factor Im is related to spliceosomal SR proteins and can be reconstituted in vitro from recombinant subunits. *Mol Cell* **1**: 243–253
Scorilas A, Black MH, Talieri M and Diamandis EP (2000) Genomic organization, physical mapping, and expression analysis of the human protein arginine methyltransferase 1 gene. *Biochem Biophys Res Commun* **278**: 349–359
Scorilas A, Kyriakopoulou L, Yousef G, Ashworth L, Kwamie A and Diamandis E (2001) Molecular cloning, physical mapping and expression analysis of a novel gene, BCL2L12, encoding for a proline-rich protein with a highly conserved BH2 domain of the Bcl-2 family. *Genomics* **73**
Screaton GR, Caceres JF, Mayeda A, Bell MV, Plebanski M, Jackson DG, Bell JI and Krainer AR (1995) Identification and characterization of three members of the human SR family of pre-mRNA splicing factors. *Embo J* **14**: 4336–4349
Serov SF and Sorbin LH (1973) Histological typing of ovarian tumors. No.9. Geneva:. *World Helth Organization* **17**
Spritz RA, Strunk K, Surowy CS, Hoch SO, Barton DE and Francke U (1987) The human U1-70K snRNP protein: cDNA cloning, chromosomal localization, expression, alternative splicing and RNA-binding. *Nucleic Acids Res* **15**: 10373–10391
Stickeler E, Kittrell F, Medina D and Berget SM (1999) Stage-specific changes in SR splicing factors and alternative splicing in mammary tumorigenesis. *Oncogene* **18**: 3574–3582
Surowy CS, Hoganson G, Gosink J, Strunk K and Spritz RA (1990) The human RD protein is closely related to nuclear RNA-binding proteins and has been highly conserved. *Gene* **90**: 299–302
Takagaki Y, Ryner LC and Manley JL (1989) Four factors are required for 3′-end cleavage of pre-mRNAs. *Genes Dev* **3**: 1711–1724
Takagaki Y, MacDonald CC, Shenk T and Manley JL (1992) The human 64-kDa polyadenylylation factor contains a ribonucleoprotein-type RNA binding domain and unusual auxiliary motifs. *Proc Natl Acad Sci USA* **89**: 1403–1407
Takezaki N (1998) Tie trees generated by distance methods of phylogenetic reconstruction. *Mol Biol Evol* **15**: 727–737
Tanner S, Stagljar I, Georgiev O, Schaffner W and Bourquin JP (1997) A novel SR-related protein specifically interacts with the carboxy-terminal domain (CTD) of RNA polymerase II through a conserved interaction domain. *Biol Chem* **378**: 565–571
Vellard M, Sureau A, Soret J, Martinerie C and Perbal B (1992) A potential splicing factor is encoded by the opposite strand of the trans-spliced c-myb exon. *Proc Natl Acad Sci USA* **89**: 2511–2515
Wang J, Xiao SH and Manley JL (1998) Genetic analysis of the SR protein ASF/SF2: interchangeability of RS domains and negative control of splicing. *Genes Dev* **12**: 2222–2233
Webb CP, Van Aelst L, Wigler MH and Woude GF (1998) Signaling pathways in Rasmediated tumorigenicity and metastasis. *Proc Natl Acad Sci USA* **95**: 8773–8778
Yousef GM, Chang A, Scorilas A and Diamandis EP (2000a) Genomic organization of the human kallikrein gene family on chromosome 19q 13.3-q 13.4. *Biochem Biophys Res Commun* **276**: 125–133
Yousef GM, Scorilas A and Diamandis EP (2000b) Genomic organization, mapping, tissue expression, and hormonal regulation of trypsin-like serine protease (TLSP PRSS20), a new member of the human kallikrein gene family. *Genomics* **63**: 88–96
Yousef GM, Scorilas A, Magklara A, Soosaipillai A and Diamandis EP (2000c) The KLK7 (PRSS6) gene, encoding for the stratum corneum chymotryptic enzyme is a new member of the human kallikrein gene family – genomic characterization, mapping, tissue expression and hormonal regulation. *Gene* **254**: 119–128
Yousef GM, Scorilas A, Jung K, Ashworth LK and Diamandis EP (2001) Molecular cloning of the human kallikrein 15 gene (KLK15). Up-regulation in prostate cancer. *J Biol Chem* **276**: 53–61
Yuryev A, Patturajan M, Litingtung Y, Joshi RV, Gentile C, Gebara M and Corden JL (1996) The C-terminal domain of the largest subunit of RNA polymerase II interacts with a novel set of serine/argine-rich proteins. *Proc Natl Acad Sci USA* **93**: 6975–6980
Zahler AM, Neugebauer KM, Stolk JA and Roth MB (1993) Human SR proteins and isolation of a cDNA encoding SRp75. *Mol Cell Biol* **13**: 4023–4028

 © 2001 Cancer Research Campaign
